# Incidence of neoplasias and effectiveness of postoperative surveillance endoscopy for patients with ulcerative colitis: comparison of ileorectal anastomosis and ileal pouch-anal anastomosis

**DOI:** 10.1186/s12957-016-0833-5

**Published:** 2016-03-09

**Authors:** Hiroaki Ishii, Keisuke Hata, Junko Kishikawa, Hiroyuki Anzai, Kensuke Otani, Koji Yasuda, Takeshi Nishikawa, Toshiaki Tanaka, Junichiro Tanaka, Tomomichi Kiyomatsu, Kazushige Kawai, Hiroaki Nozawa, Shinsuke Kazama, Hironori Yamaguchi, Soichiro Ishihara, Eiji Sunami, Joji Kitayama, Toshiaki Watanabe

**Affiliations:** Department of Surgical Oncology, Faculty of Medicine, The University of Tokyo, Tokyo, 113-0033 Japan

**Keywords:** Ulcerative colitis, Ileorectal anastomosis, Ileal pouch anal anastomosis, Postoperative surveillance endoscopy, Postoperative dysplasia/cancer

## Abstract

**Background:**

The incidence of neoplasia after surgery has not been sufficiently evaluated in patients with ulcerative colitis (UC), particularly in the Japanese population, and it is not clear whether surveillance endoscopy is effective in detecting dysplasia/cancer in the remnant rectum or pouch. The aims of this study were to assess and compare postoperative development of dysplasia/cancer in patients with UC who underwent ileorectal anastomosis (IRA) or ileal pouch-anal anastomosis (IPAA) and to evaluate the effectiveness of postoperative surveillance endoscopy.

**Methods:**

One hundred twenty patients who received postoperative surveillance endoscopy were retrospectively reviewed for development of dysplasia/cancer in the remnant rectal mucosa or pouch.

**Results:**

Three hundred seventy-nine endoscopy sessions were conducted for 30 patients after IRA, while 548 pouch endoscopy sessions were conducted for 90 patients after IPAA. In the IRA group, 5 patients developed dysplasia/cancer during postoperative surveillance and in all cases, neoplasia was detected at an early stage. In the IRA group, no patient developed neoplasia within 10 years of diagnosis; the cumulative incidence of neoplasia after disease onset was 7.2, 12.0, and 23.9 % at 15, 20, and 25 years, respectively. In one case after stapled IPAA, dysplasia was found at the ileal pouch; a subsequent 9 endoscopy sessions in 8 years did not detect any dysplasia. Neoplasia was found more frequently during postoperative surveillance in the IRA group than in the IPAA group (*p* = .0028). The cumulative incidence of neoplasia after IRA was 3.8, 8.7, and 21.7 % at 10, 15, and 20 years, respectively, and that after IPAA was 1.6 % at 20 years.

**Conclusions:**

The cumulative incidence of neoplasia after IPAA was minimal. Those who underwent IRA had a greater risk of developing neoplasia than those who underwent IPAA, although postoperative surveillance endoscopy was able to detect dysplasia/cancer at an early stage. IRA can be the surgical procedure of choice only in selected cases in which it would be of benefit to the patient, with more careful surveillance.

## Background

Cancer development in the ileal pouch and the remnant rectum has been the concern after surgical resection for patients with ulcerative colitis (UC) [1, 2]. Patients who underwent ileo-rectal anastomosis (IRA) are known to be at risk for the development of rectal cancer and require surveillance colonoscopy as those who have not received surgery [3]. BSG guidelines suggest the way of pouch surveillance after IPAA with admitting that there is no clear evidence for pouch surveillance [4]. However, the effectiveness of surveillance colonoscopy for patients with UC remains unclear.

Ileorectal anastomosis (IRA) used to be the surgical procedure for UC, which includes a total abdominal colectomy with anastomosis between the ileum and the rectum [5]. The major disadvantage of IRA is that risk of recurrent proctitis and neoplasia transformation remained in the remnant rectum. In 1978, Parks et al. [6] reported conducting a total proctocolectomy with ileal pouch-anal anastomosis (IPAA). Since then, IPAA has become the standard surgical treatment for patients with UC in most cases.

It has been reported that the risk of postoperative neoplasia is lower in patients with UC receiving IPAA due to excision of the rectum than in those receiving IRA [1, 2, 7–12]. However, cases of dysplasia/cancer have been reported even after IPAA; thus, IPAA does not completely eliminate the risk of neoplasia transformation [1, 2, 12–20]. Postoperative neoplasias have been detected at the anal transitional zone (ATZ) or in the pouch. Prior neoplasia was reported to be a risk factor for postoperative dysplasia/cancer development [1, 2, 13, 14, 21].

Kariv et al. [1] reported that the cumulative incidence of pouch neoplasia after IPAA was 1.3 and 4.2 % at 10 and 20 years, respectively, and Derikx et al. [2] reported a cumulative incidence of 2.0 and 6.9 % at 10 and 20 years, respectively. There are two major methods of reconstruction in IPAA: hand-sewn IPAA with mucosectomy and stapled IPAA without mucosectomy. There has been debate regarding the relative oncological risks of these techniques. There have been many reported cases in which stapled IPAA was selected with neoplasia as a surgical indication [1, 2, 19, 20, 22, 23]. On the other hand, Sagayama et al. [21] reported that the incidence of dysplasia in the mucosectomy area was as high as 4.4 %; therefore, they recommended performing hand-sewn IPAA with mucosectomy.

IPAA has replaced IRA for most UC surgeries due to postoperative inflammation and the risk of neoplasia development in the remnant rectum with IRA. However, IRA has several advantages over IPAA in terms of anal and sexual functions and fecundity and thus, IRA is currently considered in selected cases [7–10]. In particular, laparoscopic IRA may decrease postoperative adhesions of the fallopian tubes, increasing the probability of spontaneous pregnancy [24]. In selecting IRA for these patients, it is vital to clarify the risk of neoplasia development after IRA and the effectiveness of postoperative surveillance endoscopy, particularly whether it can detect neoplasia at an early stage. In some studies of IRA, no patient developed dysplasia/cancer within 10 years of diagnosis [9–11, 25, 26], although the risk of neoplasia transformation increased with longer follow-up [9–11]. Most patients who developed cancer after IRA presented at an advanced stage, when the prognosis was poor [9–11, 27]. There are no guidelines regarding surveillance after IRA, and the effectiveness of early detection of neoplasia is unclear.

We previously reported a postoperative surveillance cohort in 2011 with data until 2008 [28] and have updated the data with an additional 6 years of follow-up. We have shown a relatively high incidence of neoplasia in IRA series [29]. The aims of the current study were to assess and compare postoperative development of dysplasia/cancer for patients with UC between the two surgical procedures and to evaluate the effectiveness of postoperative surveillance endoscopy in our series.

## Methods

### Patients

One hundred forty-four patients with UC underwent either IRA or IPAA in our institute between 1965 and 2014. Of these, 24 patients were excluded from the study because they did not receive endoscopy after surgery. For UC patients with coexisting colorectal cancer or dysplasia, hand-sewn IPAA has been preferentially performed in our institution. A total of 120 patients were retrospectively reviewed for development of dysplasia/cancer in the remnant rectal mucosa or pouch, based on endoscopic and pathological findings. This study was carried out with the approval of the Ethical Review Board of The University of Tokyo.

### Surveillance endoscopy

Surveillance endoscopy was conducted annually in most cases. In addition to conventional observation, the indigo carmine dye-spraying method was performed for better visualization of mucosal lesions. Targeted biopsy specimens were taken from lesions suggestive of dysplasia, and random biopsy specimens were taken from apparently normal flat mucosa in the remnant rectum after IRA and from those in the ATZ area or pouch after IPAA. Dysplasia was graded according to Riddell’s classification [30] into high-grade dysplasia (HGD) and low-grade dysplasia (LGD).

### Statistical analyses

For analysis of clinicopathological variables, unpaired *t* tests or Wilcoxon rank-sum tests were used for comparison of continuous variables and the Pearson chi-square test and Fisher’s exact test (if expected cell counts were <5) for categorical variables. The cumulative incidence of neoplasia after surgery was calculated using a Kaplan-Meier curve. A log-rank test was used to compare incidences between the surgical procedures. All analyses were performed with JMP 10.0 software (SAS Institute Japan, Tokyo, Japan), and differences were considered statistically significant at *p* < .05.

## Results

A total of 927 endoscopy sessions after surgery were conducted for 120 patients with UC who underwent either IRA or IPAA (median 5 times, range 1–34). These included 379 endoscopy sessions for 30 patients after IRA and 548 pouch endoscopy sessions for 90 patients after IPAA. Patient characteristics are shown in Table 1.

**Table 1:** Patient characteristics

	IPAA	IRA	*p* value
	(*n* = 90)	(*n* = 30)	
Gender (male/female)	52/38	18/12	.83
Age at surgery (years)	38.7 ± 14.7^a^	36.4 ± 14.6^a^	.48
Disease duration at surgery (years)	10.3 ± 7.8^a^	5.5 ± 4.3^a^	.0017
Indication for surgery			
Cancer/dysplasia	27	1	.0022
Refractory	63	29	
Follow-up time (years)	10.0 (0.2–22.9)^b^	18.0 (2.3–44.1)^b^	<.0001
Total disease duration (years)	20.3 ± 9.0^a^	23.4 ± 9.7^a^	.21

*IPAA* ileal pouch-anal anastomosis, *IRA* ileorectal anastomosis

^a^Mean ± standard deviation

^b^Median (range)

IRA was the surgical procedure of choice before the 1980s in our institute mainly for medically refractory UC. In the only case of IRA performed for colon cancer, bowel obstruction due to the lesion had led to perforation of the ileum and a prior ileostomy, and it was difficult in this case to perform a total proctocolectomy because of the peritoneal adhesion. Most cases of dysplasia/cancer were in the IPAA group, and the disease duration at surgery in this group was longer than that in the IRA group (*p* = .0017). When cases of dysplasia/cancer were excluded and only refractory cases compared, there was no significant difference in disease duration at surgery between the IPAA and IRA groups (*p* = .0801). There were no significant differences in gender or age at surgery (*p* = .83, *p* = .48, respectively). Postoperative follow-up duration in the IRA group was longer than that in the IPAA group (*p* < .0001). There was no significant difference in total disease duration between the groups (*p* = .21).

Five patients who underwent IRA developed dysplasia or cancer during postoperative surveillance (Table 2). HGD was detected in the remnant rectal mucosa in 2 cases and LGD in 3 cases at the time of surveillance colonoscopy. Of these, 4 patients underwent surgical resection, including hand-sewn IPAA in 2 cases, abdominoperineal resection in 1 case, and transanal resection in 1 case. The other patient continued endoscopic follow-up without HGD being detected thereafter and ultimately discontinued surveillance due to age. Final pathological diagnoses for the surgical specimens were carcinoma with submucosal invasion in 2 cases and HGD in 2 cases. In 1 case with stapled IPAA, dysplasia was initially found in the ileal pouch, while subsequent 9 endoscopy sessions over 8 years did not detect dysplasia. In 1 patient who had undergone hand-sewn IPAA due to colitis-associated cancer, HGD was detected in the ileal pouch 2 years after IPAA. This patient underwent pouch excision, and the lesion proved to be a recurrence via dissemination rather than a newly developed neoplasia. In the present study, postoperative development of neoplasia was found mainly in refractory cases, with only 1 case of recurrence of preoperative dysplasia/cancer.

**Table 2:** Cases with neoplasia during postoperative surveillance

	Age at onset (years)	Age at first surgery (years)	Indication	Surgical technique	Age at second surgery (years)	Surgical technique	Neoplasia at surveillance	Final TNM or dysplasia grade	Follow-up from last surgery
1	29	33	Intractable	IRA	41	APR	HGD	T1N0M0	23 years, no rec
2	57	58	Intractable	IRA	77	IPAA	LGD	T1N0M0	7 years, no rec
3	22	24	Intractable	IRA	47	IPAA	LGD	HGD	8 years, no rec
4	42	43	Severe/emergency	IRA	56	TAR	HGD	HGD	4 years, no rec
5	56	58	Intractable	IRA			LGD	–	5 years, LGD
6	54	57	Intractable	IPAA			LGD	–	8 years, NEG

In one patient, HGD was detected in the ileal pouch 2 years after IPAA, which proved to be a recurrence via dissemination

*IRA* ileorectal anastomosis, *IPAA* ileal pouch-anal anastomosis, *APR* abdominoperitoneal resection, *TAR* transanal resection, *HGD* high-grade dysplasia, *LGD* low-grade dysplasia, *NEG* negative for dysplasia, *rec* recurrence

**Fig. 1:**
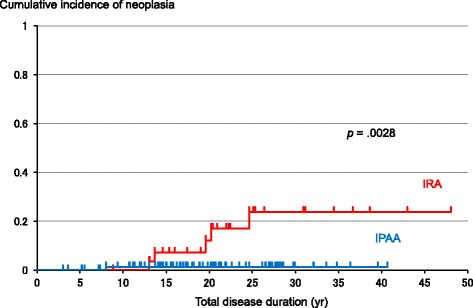
Cumulative incidence of neoplasia by surgical technique. Cumulative incidence calculated using the Kaplan-Meier method; *p* value calculated using the log-rank test. *IRA* ileorectal anastomosis, *IPAA* ileal pouch-anal anastomosis

Cumulative incidences of neoplasia by surgical technique are shown in Fig. 1. The cumulative incidence of neoplasia after disease onset in the IRA group was 7.2, 12.0, and 23.9 % at 15, 20, and 25 years, respectively. No patient developed dysplasia/cancer within 10 years of diagnosis. Neoplasias were found more frequently in the IRA group than in the IPAA group (*p* = .0028). When cases of dysplasia/cancer were excluded and only refractory cases compared, the cumulative incidence of neoplasia after disease onset in the IRA group was 7.4, 12.6, and 24.6 % at 15, 20, and 25 years, respectively, and the difference between the IRA and IPAA group remained the same (*p* = .0227). The cumulative incidence was also estimated from the point of surgery. The cumulative incidence of neoplasia after IRA was 3.8, 8.7, and 21.7 % at 10, 15, and 20 years, respectively, and that after IPAA was 1.6 % at 20 years.

## Discussion

In the present study, the cumulative incidence of dysplasia/cancer in the Japanese patients with UC who underwent IRA was 7.2, 12.0, and 23.9 % at 15, 20, and 25 years after disease onset, respectively. No patient developed dysplasia/cancer within 10 years of diagnosis. On the other hand, dysplasia was found in only 1 patient out of 90 who underwent IPAA, and only during one surveillance session. Thus, neoplasias were found substantially more frequently in the IRA group than in the IPAA group. All of the neoplasias were successfully detected in early stages (stage 0 or I) with annual surveillance colonoscopy. Although patients with colitis-associated cancer have poorer prognosis than those with sporadic cancer in stage III, the prognosis is comparable in stages I and II [31]. Therefore, we believe that our postoperative surveillance program has effectively detected neoplasia at an early stage among patients who underwent IRA. IPAA is generally recommended for patients with UC based on the risk of cancer in the remnant rectum or ileal pouch. However, these results demonstrate that IRA with vigilant surveillance can be a viable alternative for those who hope to become pregnant or to preserve anal and sexual functions.

Kariv et al. [1] reported that the cumulative incidence of pouch neoplasia after IPAA was 1.3 and 4.2 % at 10 and 20 years, respectively. Derekx et al. [2] reported 2.0 and 6.9 % at 10 and 20 years, respectively. The cumulative incidence of neoplasia in our series was 1.6 % 20 years after IPAA, lower than that in the previous studies. This may reflect the choice of surgical procedure in our institute. Stapled IPAA is sometimes performed for patients with dysplasia/cancer due to the simplicity of its surgical procedure and to preserve postoperative anal function [1, 2, 19, 20, 22, 23]; however, neoplasias have frequently been detected in the ATZ area after stapled IPAA [1, 2, 19, 20]. We have basically performed hand-sewn IPAA with mucosectomy for patients with dysplasia/cancer, which may account for the decreased development of postoperative neoplasia in our series.

In previous reports, no IRA patient developed rectal cancer within 10 years of diagnosis [9–11, 25, 26], although the risk of neoplasia transformation increased with longer follow-up [9–11]. Andersson et al. [10] reported a cumulative cancer risk of 0 % at 10 years, 2.1 % at 20 years, and 8.7 % at 25 years. Similarly, Baker et al. [11] reported 0 % at 10 years, 6 % at 20 years, and 18 % at 35 years, while da Luz Moreira reported 2 % at 10 years and 14 % at 20 years [9]. Our results are consistent with these reports and also confirm that development of neoplasia is significantly more frequent after IRA than after IPAA.

In our series, all dysplasias/cancers were successfully detected at early stages (stages 0 and I) with postoperative surveillance endoscopy and all patients survived without recurrence after additional treatment, although previous studies have reported that patients with cancer after IRA had poor prognosis [9–11, 27]. Notably, postoperative development of neoplasia in the present study was found in refractory cases. Therefore, it is important to conduct surveillance endoscopy after IRA regardless of surgical indication. In recent years, IRA has been performed for selected cases in consideration of anal function, sexual function, and fertility [7–10]. Postoperative surveillance is particularly important in such cases.

There were several limitations of this study. First, the sample size was small. Second, this was a retrospective study. Third, longer follow-up may be needed since patients with UC are young at disease onset. Therefore, further research with a larger number of patients and longer follow-up will be needed to confirm the validity of these results.

## Conclusions

The present study demonstrated that the cumulative incidence of neoplasia after IPAA is minimal. Those who underwent IRA had higher risk of neoplasia development than those who underwent IPAA, although postoperative surveillance enabled us to detect dysplasia/cancer at an early stage. IRA can be the surgical procedure of choice in selected cases in which it would be of benefit to the patient, with more careful surveillance.
